# Knowledge, attitudes, and practices regarding pressure injury prevention among prehospital emergency medical personnel: a cross-sectional survey

**DOI:** 10.3389/fpubh.2026.1781417

**Published:** 2026-04-17

**Authors:** Yan Xue, JingBo Zhu, DanYan Wu, LiPing Cai, ChenYe Yuan, Ping Zhou, Jing Wang

**Affiliations:** 1 Gastrointestinal Surgery, Wuxi No 2 People's Hospital, Wuxi, China; 2 Wound Care Clinic, Wuxi No 2 People's Hospital, Wuxi, China; 3 Emergency Room, Wuxi No 2 People's Hospital, Wuxi, China

**Keywords:** attitude, knowledge, practice, prehospital emergency care, pressure injury

## Abstract

**Objective:**

To assess the current knowledge, attitudes, and practices regarding pressure injury (PI) among pre-hospital emergency medical personnel and analyze the influencing factors, aiming to provide evidence for the development of relevant training and educational programs in emergency centers.

**Methods:**

A convenience sampling method was used to select pre-hospital emergency medical personnel from eight cities in Jiangsu Province as study participants between January and March 2025. Data were collected using a general information questionnaire and a PI knowledge-attitudes-practices (PI KAP) questionnaire. Univariate analysis and multiple linear regression were performed to explore the influencing factors. The Strengthening the Reporting of Observational Studies in Epidemiology (STROBE) guidelines were applied.

**Results:**

A total of 251 valid questionnaires were collected, with an effective response rate of 95.8%. The total score for the pre-hospital emergency medical personnel's PI KAP questionnaire was 121.2 ± 19.1, corresponding to a score rate of 78.2%. Among the participants, 51.8% exhibited suboptimal knowledge of PI prevention, 68.5% held a positive attitude toward PI prevention, and 56.6% reported that PI prevention practices were not consistently implemented. Multivariable linear regression analysis showed that female, longer work experience, being a nurse, and senior professional title were associated with higher total PI-related KAP scores or higher scores in specific domains, whereas a master's degree or higher and no participation in PI-related training were associated with lower total or domain-specific scores. Notably, no participation in PI-related training was associated with lower total KAP and lower scores across the knowledge, attitude, and practice domains.

**Conclusion:**

Prehospital emergency medical personnel exhibited a positive attitude toward PI prevention, but their knowledge and practices did not reach the desired level. It is recommended to enhance PI-related training, particularly in knowledge dissemination and the implementation of practices, to further improve the PI prevention capabilities of pre-hospital emergency medical personnel, ensuring skin safety for patients during transport.

## 1 Introduction

Pressure injury (PI) refers to localized damage to the skin and/or underlying tissue, typically occurring at areas of bony prominences or in association with medical devices or other objects (1. The significance of PI has been widely acknowledged worldwide. They are not only regarded as a key component of hospital patient safety management but also serve as a critical indicator for evaluating patient safety and nursing quality in healthcare institutions. The latest guidelines published by the National Pressure Injury Advisory Panel (NPIAP) and the European Pressure Ulcer Advisory Panel (EPUAP) highlight that “patients during tr)ansport” are one of the high-risk groups for PIs and emphasize that “prevention is better than treatment” (1).

Compared to the relatively stable and resource-abundant environment within hospitals, pre-hospital emergency transport is characterized by high dynamics and complexity. The transport process is subject to various unfavorable factors, such as limited space, bumpy roads, and concentrated equipment (2). Although pressure-redistribution foam mattresses (3), prophylactic dressings (4), and monitoring devices (5) have been shown to help mitigate pressure exposure, surveys indicate that these measures are rarely used during prehospital transport (2). Consequently, prevention in this setting often focuses on risk identification and handover reporting. Because patients are often required to remain fixed on a stretcher or spinal board for extended periods, which makes timely repositioning difficult and further increases the risk of PI. At the same time, some patients require maintenance of airway, breathing, and circulatory stability during transport. Medical interventions such as endotracheal intubation, cervical collars, and monitoring tubes can increase the risk of pressure injuries at the sites of pressure and medical device-related pressure injuries (MDRPIs) (6, 7). Existing studies have shown that approximately 5.2%−7.9% of patients already have varying degrees of PIs or are at high risk of skin pressure upon arrival at the hospital emergency department (2, 6), indicating that some PIs may have developed or worsened during the pre-hospital phase. In the prehospital setting, emergency medical personnel may not always be able to accurately ascertain the duration of pressure exposure before transport, which can introduce uncertainty into PI risk assessment and the selection of preventive measures. As PIs can develop rapidly within 2 h (2), failure to fully identify and assess PI upon entry to a healthcare facility may delay preventive and therapeutic interventions, leading to further deterioration of the injury. This significantly increases the risk of adverse outcomes such as pain, infection, and other complications (8, 9), while also prolonging hospital stays, increasing readmission rates, and raising mortality rates, imposing a heavy economic burden on the healthcare system (9–11). Therefore, pre-hospital emergency transport is not only a critical link between the pre-hospital and in-hospital phases but also a key point for the prevention and early intervention of PIs.

Prehospital transport-related PI research has largely focused on epidemiology and associated risk factors. Evidence suggests that PI during transport is not uncommon, with frequently affected sites including the sacral coccygeal region, heels, ankles and buttocks. Reported risk factors include transport duration and frequency, transport equipment and environmental conditions, and patient-related characteristics (12). In addition, MDRPIs during transport have received increasing attention. Observational studies indicate that immobilization devices such as cervical collars and spine boards may generate substantial localized pressure and contribute to characteristic injury patterns at specific anatomical sites (6). By contrast, evidence on prehospital emergency personnel's knowledge, attitudes, and practices (KAP) regarding PI prevention remains limited. Only a few studies have described their PI-related knowledge and attitudes (13), highlighting an ongoing gap in provider-level evidence.

In the high-risk context of pre-hospital emergency transport, pre-hospital emergency medical personnel (including doctors and nurses) are the key executors of PI risk assessment and prevention interventions, forming the first line of defense for PI prevention during transport. The KAP levels of medical personnel regarding PI directly affect the timeliness of risk assessment, the implementation of preventive measures, and the ongoing attention to the patient's skin condition (14). However, prehospital transport care is a multidimensional and comprehensive process. In addition to life-support interventions, it encompasses ongoing assessment and monitoring, positioning management, line and device management, as well as communication and handover. Under time-critical and resource-constrained conditions, providers often need to balance competing priorities across these tasks (2, 6). Consequently, the standardized implementation of PI risk assessment and preventive measures during transport may be challenging. Even when some medical personnel possess a certain level of knowledge about PI, the lack of standardized procedures, time constraints, inadequate equipment, and poor team communication under multiple pressures may result in their actions not fully reflecting their knowledge, leading to a phenomenon of “knowledge-action gap”. Therefore, systematically assessing the capabilities and shortcomings of pre-hospital emergency medical personnel in PI prevention from a KAP perspective is of significant importance for advancing skin safety management during transport.

Research has shown that the key to reducing the occurrence of PI lies in the attitude and involvement of medical personnel (15). Currently, research on the KAP of PI primarily focuses on nurses within hospital settings, such as those in general wards (16), ICUs (17), and operating rooms (18), with limited attention given to the specific group of pre-hospital emergency medical personnel. Therefore, this study utilizes a specialized questionnaire developed and validated by our research team to conduct a cross-sectional survey (19). The aim is to assess the current status and influencing factors of PI KAP among pre-hospital emergency medical personnel, with the goal of providing a basis for emergency center managers to design scientific and effective training programs and optimize the quality of emergency care.

## 2 Methods

### 2.1 Study design and participants

This was a cross-sectional study conducted from January to March 2025, in which pre-hospital emergency medical personnel from eight cities in Jiangsu Province were selected as study participants using convenient sampling. This study was approved by the Ethics Committee of Wuxi No 2 People's Hospital (ethics no 2024Y-132). Inclusion criteria were: (1) age ≥ 18 years; (2) pre-hospital emergency medical personnel; (3) possession of a valid medical professional qualification (doctor/nurse); and (4) at least one year of work experience. Exclusion criteria included individuals who refused to participate in the study. To enhance the clarity, accuracy, and transparency of the report, this study adhered to the Strengthening the Reporting of Observational Studies in Epidemiology (STROBE) guidelines.

The sample size was determined using Green's rule of thumb for multiple regression (20), which specifies that the minimum sample size should be N ≥ 50 + 8m, where m is the number of independent variables. For 10 independent variables, the minimum required sample size was 130. To account for an expected 20% non-response rate, the sample size was adjusted to 163. Ultimately, 251 valid participants were included, which was considered adequate for the planned statistical analyses. This study chose a larger sample size primarily to ensure adequate statistical power, particularly for subgroup analyses, and to enhance the representativeness of the sample, thereby increasing the stability and reliability of the results.

### 2.2 Data collection tools

#### 2.2.1 General information questionnaire

Designed by the research team, the questionnaire included items on gender, age, years of work experience, educational level, profession, professional title, whether the participant was a manager, whether they had transported PI patients, and whether they had received PI-related training.

#### 2.2.2 The pre-hospital emergency medical personnel's PI KAP questionnaire

The PI KAP questionnaire for prehospital emergency medical personnel was developed by the research team in 2024 (19). It was developed based on the knowledge-attitude-practice framework through literature review, expert consultation, and a pilot survey. The final items were determined after item analysis and reliability and validity testing. The questionnaire consists of three dimensions: knowledge, attitudes, and practices, with 9, 11, and 11 items, respectively, totaling 31 items. All items are scored using a 5-point Likert scale, with a total score range from 31 to 155. The total score range for the knowledge, attitude, and practice dimensions is 9–45, 11–55, and 11–55, respectively. Specifically, the knowledge items were scored from 1 (do not know) to 5 (very familiar), the attitude items from 1 (strongly disagree) to 5 (strongly agree), and the practice items from 1 (never) to 5 (always). The scores of individual items within each dimension were summed to obtain the total score for that dimension. The Cronbach's α coefficient for the overall questionnaire is 0.959, while the split-half reliability coefficients for each dimension are 0.885, 0.879, and 0.936, respectively. The questionnaire demonstrated good reliability and validity in the original validation study. A final open-ended item was included in the questionnaire, “What difficulties do you encounter in implementing PI prevention for patients during transport?” This question provided eight predefined options along with an “Other” option, allowing participants to provide additional responses under the “Other” category. Participants were allowed to select all predefined options, and the results are not mutually exclusive. Responses to this question were not included in the total score. The full questionnaire is provided in Supplementary File 1.

In an Australian study on PI prevention knowledge, a sufficient knowledge threshold was set at ≥90% (21). In Fang et al.'s study, a satisfactory attitude score for PI prevention was defined as ≥75% (22). A Chinese study set the threshold for satisfactory PI prevention practice performance at >85% (23). Due to the lack of consistent evidence regarding appropriate cut-off scores for knowledge, attitude, and practice tools, this study adopts a cut-off value of 80%, which is consistent with the threshold set by Jiang et al. (21). Based on this criterion, participants' levels were categorized according to the total scores of each domain. Specifically, a score reaching or exceeding 80% of the maximum possible total score for each dimension was defined as good knowledge, a positive attitude, and active practice, whereas scores below this threshold were considered as below expectation.

### 2.3 Data collection

The researchers conducted the survey using an electronic questionnaire. Prior to the survey, consent and cooperation were obtained from the director of the emergency center. The medical personnel being surveyed were invited to participate via a formal notification, which included information about the study's purpose and significance. They were asked to complete the questionnaire using their mobile phones or computers. The questionnaire included a standardized instruction that explained the purpose, significance, and completion method of the survey. All questionnaires were anonymous, and a commitment was made to keep the survey results confidential. To ensure the authenticity and reliability of the results, each mobile phone or IP address was allowed to submit the questionnaire only once, and all options were mandatory. A total of 262 questionnaires were collected, with 251 valid ones, resulting in an effective response rate of 95.8%. All data were entered by two individuals to ensure accuracy.

### 2.4 Data analysis

Data analysis was conducted using SPSS 26.0 statistical software. For categorical data, frequency and percentage were used for description. For continuous data that followed a normal distribution, the mean ± standard deviation was used for description. Independent samples *t*-test was used for comparison between two groups, and analysis of variance (ANOVA) was used for comparison among multiple groups. If the assumption of homogeneity of variances was not met, the rank sum test was applied. For continuous data that did not follow a normal distribution, the median and interquartile range (IQR) were used for description, and non-parametric tests were employed for analysis. Multivariable linear regression analysis was used to identify the influencing factors of PI KAP. Variables with *p* < 0.20 in the univariate analysis were included in the multivariable linear regression model using a backward conditional approach, with statistical significance set at *p* < 0.05. The exponential beta coefficient and 95% confidence intervals (CIs) were reported. For open-ended questions, responses to the “Other” option were qualitatively analyzed and categorized into themes. The frequency and percentage of each theme and predefined option were calculated.

## 3 Results

### 3.1 Participants characteristics

A total of 262 prehospital emergency medical personnel were enrolled, of whom 251 completed the questionnaire, resulting in an effective response rate of 95.8%. Eleven participants were excluded due to incomplete or invalid responses. The participants had an average age of 36.6 years, with the majority aged between 26 and 35 years (49.8%). Most of the participants were female (61.4%). The majority had 10–20 years of work experience (37.1%), and most had an educational level of a bachelor's degree (83.3%). The majority of the transport medical personnel were nurses (63.7%). Among all participants, only a small proportion were managers (13.5%), more than half had transported PI patients (67.3%), but only one-third had received PI-related training (36.3%). The detailed demographic information of the participants is shown in Table 1.

**Table 1:** Characteristics of the participants (*n* = 251).

Variable	Categories	Frequency (%)
Gender	Male	97 (38.6)
	Female	154 (61.4)
Age (years)	≤ 25	10 (4.0)
	26 ~ ≤ 35	125 (49.8)
	36 ~ ≤ 45	90 (35.9)
	> 45	26 (10.3)
Working seniority (years)	< 5	44 (17.5)
	5 ~ < 10	78 (31.1)
	10 ~ < 20	93 (37.1)
	≥ 20	36 (14.3)
Education	Specialty degree	24 (9.6)
	Bachelor's Degree	209 (83.3)
	Master's degree or higher	18 (7.1)
Profession	Doctor	91 (36.3)
	Nurse	160 (63.7)
Professional title (doctor)	Junior	35 (13.9)
	Intermediate	39 (15.5)
	Senior	17 (6.8)
Professional title (nurse)	Staff Nurse	13 (5.2)
	Charge Nurse	68 (27.1)
	Associate Chief Nurse and above	79 (31.5)
Managerial role	Yes	34 (13.5)
	No	217 (86.5)
Transferred PI Patients	Yes	169 (67.3)
	No	82 (32.7)
Participation in PI-related training	Yes	91 (36.3)
	No	160 (63.7)

### 3.2 PI KAP scores among prehospital emergency medical personnel

In this survey, prehospital emergency medical personnel involved in patient transport achieved a total score of 121.2 ± 19.1 on the PI KAP questionnaire, corresponding to a score rate of 78.2%. The knowledge subscale score was 34.1 ± 6.8 (score rate: 75.7%), the attitude subscale score was 46.6 ± 6.5 (score rate: 84.7%), and the practice subscale score was 40.6 ± 9.3 (score rate: 73.8%). The detailed scores for each item, as well as the domain-level classification according to the 80% cut-off values, are presented in Supplementary File 1. The original Chinese version of the questionnaire is provided in Supplementary File 2. Demographic analysis revealed significant differences in the total PI KAP questionnaire and practice scores, including gender, education, profession, and participation in PI-related training. Knowledge and attitude scores varied based on education, profession, and whether participants had received PI-related training. The detailed results are presented in Table 2.

**Table 2:** Univariate analysis of PI KAP questionnaire scores and their dimensions among prehospital emergency medical personnel (*n* = 251).

Variable	(Mean ±SD) / Median (IQR)			
	PI KAP	Knowledge dimension	Attitude dimension	Practice dimension
**Score range**	31–155	9–45	11–55	11–55
Gender				
Male	117.9 ± 21.7	35.0(27.0,38.5)	45.0(40.0,53.0)	42.0(32,0,44.0)
Female	124.0(111.8,135.5)	35.5(32.0,37.3)	47.0(44.0,53.0)	42.0(36.0,46.0)
*p*-value	0.025^1)^	0.209^1)^	0.113^1)^	0.049^1)^
Age (years)				
≤ 25	115.5(112.0,134.0)	36.0 ± 5.1	44.0(43.5,47.5)	41.1 ± 8.5
26 ~ ≤ 35	124.0(108.0,137.0)	36.0(29.0,38.5)	46.0(43.0,52.5)	43.0(34.5,46.0)
36 ~ ≤ 45	121.6 ± 19.0	35.0(28.0,37.0)	47.0(43.0,54.0)	40.7 ± 9.2
> 45	117.0 ± 19.0	33.3 ± 6.3	46.23 ± 6.9	37.4 ± 9.3
*p*-value	0.775^2)^	0.621^2)^	0.782^2)^	0.254^2)^
Working seniority (years)				
< 5	115.5 ± 19.1	31.5 ± 7.2	45.5 ± 5.9	38.6 ± 9.5
5 ~ < 10	120.3 ± 19.7	35.0(28.0,38.0)	45.7 ± 7.0	43.0(33.8,46.0)
10 ~ < 20	124.4 ± 18.3	36.0(32.0,39.0)	47.0(43.0,54.0)	43.0(36.0,47.5)
≥20	122.1 ± 18.3	34.8 ± 6.2	50.0(42.3,54.0)	39.3 ± 9.4
*p*-value	0.077^3)^	0.055^2)^	0.170^2)^	0.179^2)^
Education				
Specialty degree	121.0 ± 16.9	35.0 ± 5.7	44.8 ± 6.2	41.1 ± 7.6
Bachelor's Degree	123.0(111.0,137.0)	36.0(30.0,38.0)	47.0(43.0,53.0)	43.0(34.5,46.0)
Master's degree or higher	104.0 ± 15.7	26.9 ± 5.7	43.8 ± 6.9	33.3 ± 7.2
*p*-value	< 0.001^2)^	< 0.001^2)^	0.049^2)^	0.003^2)^
Profession				
Doctor	112.7 ± 19.4	29.0(25.0,36.0)	44.0(40.0,53.0)	38.0(30.0,44.0)
Nurse	126.5(113.3,138.8)	36.0(33.0,39.8)	47.0(43.0,53.0)	44.0(37.0,49.0)
*p*-value	< 0.001^1)^	< 0.001^1)^	0.042^1)^	< 0.001^1)^
Professional title (doctor)				
Junior	113.0 ± 19.4	27.0(24.0,34.0)	46.5 ± 6.7	38.2 ± 9.2
Intermediate	111.2 ± 19.5	30.4 ± 6.8	44.0 ± 7.1	34.0(30.0,44.0)
Senior	115.5 ± 20.3	35.0(27.0,36.5)	50.0(37.0,54.0)	38.0(33.0,43.0)
*p*-value	0.749^3)^	0.057^2)^	0.265^2)^	0.860^2)^
Professional title (nurse)				
Junior	119.2 ± 14.2	44.0(42.5,46.5)	45.1 ± 4.4	39.6 ± 8.5
Intermediate	126.3 ± 18.0	36.0(33.3,41.8)	46.5 ± 6.6	44.0(37.3,49.8)
Senior	127.1 ± 16.6	36.0(33.0,39.0)	49.0(44.0,54.0)	42.4 ± 9.0
*p*-value	0.302^3)^	0.328^2)^	0.088^2)^	0.228^2)^
Managerial Role				
Yes	121.9 ± 21.9	36.0(27.0,41.0)	50.0(43.8,54.0)	38.6 ± 11.5
No	121.1 ± 18.6	35.0(29.0,37.0)	46.0(42.0,52.3)	42.0(34.0,46.0)
*p*-value	0.834^4)^	0.291^1)^	0.092^1)^	0.335^1)^
Transferred PI patients				
Yes	121.3 ± 19.5	35.0(29.0,38.0)	47.0(42.0,53.0)	42.0(33.0,47.0)
No	121.2 ± 18.3	34.5(27.8,36.3)	46.0(43.0,53.0)	42.0(35.8,45.0)
*p*-value	0.989^4)^	0.740^1)^	0.940^1)^	0.866^1)^
Participation in PI-related training				
Yes	139.0(130.0,147.0)	37.0(33.0,44.0)	54.0(52.0,55.0)	46.0(42.0,55.0)
No	115.5(102.0,124.0)	34.0(27.0,36.0)	44.0(39.3,46.0)	39.0(33.0,44.0)
*p*-value	< 0.001^1)^	< 0.001^1)^	< 0.001^1)^	< 0.001^1)^

Variables are presented as Mean ± SD for normally distributed data and as median (IQR) for non-normally distributed data; SD, standard deviation; IQR, interquartile range.

^1)^Mann-Whitney U-test;

^2)^Kruskal-Wallis test;

^3)^one-way ANOVA;

^4)^Independent-samples t-test.

Total sample size = 251; n = 91 for Professional title (doctor); n = 160 for Professional title (nurse).

Regarding barriers to implementing PI prevention, 74.1% of participants reported insufficient PI-prevention resources (e.g., dressings, soft pillows/cushions), and 68.1% indicated that the ambulance environment was not conducive to delivering PI-prevention measures (In the “Other” category, one participant noted that “position changes during transport in the ambulance could pose safety risks,” which was categorized under this predefined option). In addition, 48.6%, 35.5%, and 48.2% of participants reported inadequate attention to the importance of PI prevention by the transport center, themselves, and accompanying family members, respectively (In the “Other” category, one participant mentioned that “family members worry about patient comfort and have poor cooperation,” which was categorized under the predefined option of “accompanying family members neglect the importance of PI prevention”). Nearly half of the participants (48.2%) noted that PI-prevention equipment increased out-of-pocket costs for families. Furthermore, 36.3% reported insufficient theoretical knowledge and practical skills related to PI prevention, and 33.1% perceived that implementing PI-prevention measures increased workload.

### 3.3 Multiple linear regression analysis of PI KAP questionnaire scores and the knowledge, attitude, and practice subscale scores among prehospital emergency medical personnel

Multivariable linear regression analysis revealed that female gender, a work experience of 10–20 years, and being a nurse were independently associated with higher total KAP scores. Work experience of 10–20 years, work experience of 20 years or more, being a nurse, and having a senior professional title (doctor) were independently associated with higher knowledge scores. Being a nurse and holding a senior professional title (nurse) were independently associated with higher attitude scores. Female gender and being a nurse were independently associated with higher Practice scores. In contrast, participants with a master's degree or higher had lower total KAP, knowledge, and practice scores, whereas those who had not received PI-related training generally had lower total KAP scores as well as lower scores across all subdomains. Detailed regression coefficients, confidence intervals, and significance levels are presented in Table 3.

**Table 3:** Multiple linear regression analysis of PI KAP questionnaire among prehospital emergency medical personnel.

Dependent variable	Dependent variable	Exp B	95% CI		*p*
			Lower	Upper	
PI KAP^1)^					
Gender	Male	1.0	–	–	–
	Female	5.454	0.628	10.280	0.027
Working seniority (years)	<5	1.0	–	–	–
	5 ~ <10	4.711	−2.309	11.731	0.187
	10 ~ <20	5.080	0.207	9.953	0.041
	≥20	3.526	−3.547	10.599	0.327
Education	Specialty degree	1.0	–	–	–
	Bachelor's Degree	1.710	−6.146	9.565	0.669
	Master's degree or higher	−18.575	−27.477	−9.674	<0.001
Profession	Doctor	1.0	–	–	–
	Nurse	13.397	8.752	18.042	<0.001
Participation in PI–related training	Yes	1.0	–	–	–
	No	−23.546	−27.513	−19.580	<0.001
Knowledge dimension^2)^					
Working seniority (years)	<5	1.0	–	–	–
	5 ~ <10	2.379	−0.094	4.852	0.059
	10 ~ <20	3.728	1.328	6.128	0.002
	≥ 20	3.295	0.348	6.243	0.029
Education	Specialty degree	1.0	–	–	–
	Bachelor's Degree	−0.496	−3.246	2.253	0.723
	Master's degree or higher	−7.652	−10.768	−4.537	<0.001
Profession	Doctor	1.0	–	–	–
	Nurse	6.195	4.626	7.764	<0.001
Professional title (doctor)	Junior	1.0	–	–	–
	Intermediate	2.153	−1.082	5.388	0.189
	Senior	3.785	0.033	7.536	0.048
Participation in PI-related training	Yes	1.0	–	–	–
	No	−4.338	−6.001	−2.675	<0.001
Attitude dimension^3)^					
Gender	Male	1.0	–	–	–
	Female	1.563	−0.097	3.224	0.065
Working seniority (years)	<5	1.0	–	–	–
	5 ~ <10	0.225	−2.189	2.639	0.855
	10 ~ <20	1.713	−0.045	3.472	0.056
	≥20	2.402	−0.022	4.825	0.052
Education	Specialty degree	1.0	–	–	–
	Bachelor's Degree	2.186	−0.566	4.938	0.119
	Master's degree or higher	−1.056	−5.037	2.926	0.602
Profession	Doctor	1.0	–	–	–
	Nurse	2.010	0.335	3.684	0.019
Professional title (nurse)	Junior	1.0	–	–	–
	Intermediate	1.452	−2.066	4.971	0.416
	Senior	2.045	0.209	3.882	0.029
Managerial role	Yes	1.0	–	–	–
	No	−1.985	−4.351	0.381	0.100
Participation in PI-related training	Yes	1.0	–	–	–
	No	−10.265	−11.372	−9.157	<0.001
Practice dimension^4)^					
Gender	Male	1.0			
	Female	2.469	0.098	4.841	0.041
Working seniority (years)	<5	1.0			
	5 ~ <10	1.806	−1.110	4.721	0.224
	10 ~ <20	2.108	−0.289	4.505	0.084
	≥20	0.641	−3.484	4.766	0.760
Education	Specialty degree	1.0	–	–	–
	Bachelor's Degree	0.089	−3.800	3.977	0.964
	Master's degree or higher	−7.885	−12.290	−3.481	0.001
Profession	Doctor	1.0	–	–	–
	Nurse	5.161	2.826	7.496	<0.001
Participation in PI-related training	Yes	1.0	–	–	–
	No	−8.889	−11.042	−6.735	<0.001

PI, pressure injury;

^1)^R^2^ = 0.498, Adjusted R^2^ = 0.488, F = 48.571, p <0.001;

^2)^R^2^ = 0.340, Adjusted R^2^ = 0.326, F = 25.197, p <0.001;

^3)^R^2^ = 0.616, Adjusted R^2^ = 0.605, F = 155.680, p <0.001;

^4)^R^2^ = 0.293, Adjusted R^2^ = 0.278, F = 20.263, p <0.001.

## 4 Discussion

This study employed a PI KAP questionnaire for prehospital emergency medical personnel involved in patient transport, which was previously developed and validated by our research team (19), to systematically assess their levels of knowledge, attitudes, and practices regarding PI prevention and the associated influencing factors. The results showed that 51.8% of participants had PI prevention knowledge levels below the desired level, 68.5% reported positive attitudes toward PI prevention, and more than half (56.6%) demonstrated insufficient preventive practices. Overall, prehospital transport personnel exhibited a pattern of relatively positive attitudes but suboptimal knowledge reserves and implementation of preventive practices, indicating substantial room for improvement in PI prevention and control within the unique context of prehospital transport.

In the knowledge domain, the score rate among prehospital emergency medical personnel involved in patient transport was 75.7%, which is comparable to findings from a study of prehospital emergency nursing staff in Sweden (13). Notably, more than half of the participants did not achieve a good level of knowledge, indicating persistent gaps in PI risk assessment, identification of risk factors, and understanding of key preventive measures. This pattern may be closely related to the clinical priorities of prehospital emergency care. During patient rescue and transport, time pressure and resource constraints often lead prehospital providers to prioritize stabilization of vital functions, particularly respiratory and circulatory status. Consequently, PI, a preventable but non-immediately life-threatening safety risk, may be assigned a lower priority and receive less systematic emphasis in training and quality management (12), resulting in insufficiently systematic and sustained reinforcement of PI-related knowledge.

Although prehospital emergency medical personnel involved in patient transport demonstrated a relatively high level of positive attitudes in this study (score rate 84.7%), their PI-prevention practice score was noticeably lower (73.8%) and was below that reported in other studies on healthcare professionals' PI-prevention practices (75.3%−86.1%) (17, 18, 24). This finding suggests a substantial attitude-practice gap in this population. To further elucidate this discrepancy, we investigated barriers to implementing PI prevention during transport, and the results revealed constraints operating at multiple levels. From the perspective of objective conditions, more than two thirds of prehospital emergency medical personnel reported insufficient availability of PI-prevention resources and indicated that the ambulance environment was not conducive to implementing preventive measures. These findings suggest that inadequate equipment provision and spatial constraints during transport constitute major structural barriers to PI prevention in the prehospital setting. Previous evidence has shown that pressure-relieving devices such as soft pillows/cushions and prophylactic dressings can effectively reduce the risk of PI development (25). However, such preventive supplies have not been systematically incorporated into standard prehospital transport equipment inventories. As a result, even personnel with positive attitudes may be unable to consistently translate intentions into effective preventive practices in routine work. At the levels of cognition and system support, some participants perceived insufficient emphasis on PI prevention from the transport center, the healthcare professionals themselves, and accompanying family members. Others expressed concern that PI-prevention supplies might increase families' financial burden or add to the workload under the high-intensity pace of prehospital transport. Collectively, these findings suggest that a shared understanding of PI risk and a systematized management consensus have not yet been established within the prehospital transport system. Limited managerial attention to PI prevention may reduce clinicians' motivation to engage in relevant training and continuous learning, thereby constraining patients' and families' awareness of PI risk and its potential adverse consequences. Taken together, these multiple factors impede the routine implementation of PI prevention in the high-risk context of prehospital transport.

Available evidence suggests that there remains considerable room to improve PI prevention during prehospital transport and the transition to emergency department care. Prophylactic dressings are simple to apply and feasible during short transports, and multiple studies indicate that they may reduce the risk of pressure-related injury at high-risk sites (26–28). Improvements in support surfaces (e.g., foam surfaces, alternating-pressure air surfaces, and reactive gel surfaces) represent more fundamental infrastructure measures. These surfaces can help mitigate sustained pressure and may be adaptable to the prehospital transport setting (29–31). Where resources allow, pressure-monitoring technologies may assist in identifying prolonged high-pressure exposure and guiding offloading, although their implementation in the prehospital setting may be constrained by cost and system-level support (3). Given the time-critical and space-limited nature of prehospital transport, transport services could prioritize incorporating lightweight, portable, and rapidly deployable pressure-relieving and protective resources (e.g., offloading cushions and prophylactic dressings) into standard equipment lists. In parallel, emergency medical service administrators could develop concise, feasible PI-prevention protocols aligned with established guidelines and tailored to prehospital workflows. These protocols can integrate risk assessment, key preventive actions, and handover documentation into routine transport processes, with implementation reinforced through targeted training and quality monitoring.

The multivariable linear regression analysis indicated that PI-related training, gender, working seniority, profession, professional title, and educational level were all independently associated with PI-related KAP among prehospital emergency medical personnel involved in patient transport. Among these variables, participation in PI-related training was one of the most consistently associated factors. Those who had not received PI-related training had significantly lower scores in the knowledge, attitude, and practice domains, suggesting that training may affect not only knowledge acquisition, but also awareness of the importance of PI prevention and the implementation of preventive practices. Compared with relatively fixed demographic characteristics such as sex and educational level, training is more likely to directly influence the translation of knowledge into attitudes and practice. However, the coverage of PI-related training was relatively low in this study, indicating that the current training system for prehospital emergency medical personnel involved in patient transport remains inadequate and warrants further improvement. In addition, the findings of this study indicated that female prehospital emergency medical personnel achieved higher scores in PI KAP than their male counterparts. Although the difference was statistically significant, its practical significance remains unclear because the median practice score was the same for male and female. Working seniority was associated with the knowledge domain, with higher knowledge scores observed among those with longer professional experience, suggesting that accumulated clinical experience may contribute to better recognition of PI risk and greater awareness of prevention. Occupation and professional title were significantly associated with PI KAP levels. Nurses generally achieved higher scores than physicians across the knowledge, attitude, and practice domains, which may be attributable to differences in primary roles and responsibilities between prehospital emergency physicians and nurses. Physicians typically assume primary responsibility for patient assessment and critical treatment decision-making during transport, prioritizing the stabilization of vital functions and overall patient safety; nurses more often ensure the implementation of prescribed interventions and provide ongoing nursing care, playing a key role in executing preventive measures such as skin assessment and positioning management. A senior physician title was associated with higher knowledge scores, whereas a senior nursing title was associated with higher attitude scores, suggesting that professional maturity and role-specific experience may facilitate the accumulation of PI-related knowledge and the development of more positive attitudes. Notably, having a postgraduate degree showed a negative association with scores in certain domains, a finding that is not entirely consistent with some previous studies (32). This finding may be related to differences in job responsibilities, fewer opportunities for hands-on practice, or more conservative self-assessment of PI prevention. However, because this study did not collect information on participants' primary job duties or their specific level of involvement in prehospital transport, the underlying mechanisms could not be examined.

Overall, PI-related KAP among prehospital emergency medical personnel involved in patient transport was associated with a range of individual and professional factors, among which PI-related training may be one of the most actionable targets for improvement. Previous studies have shown that PI prevention education and training can significantly enhance healthcare professionals' ability to identify PI risk and strengthen their preventive awareness (33–35). Given the constrained staffing and high workload in prehospital transport, some missions may be undertaken by a single physician. Therefore, emergency medical service administrators should incorporate PI-prevention content into both pre-service orientation and continuing education programs, with training content tailored to the specific demands of prehospital transport and focused on practical application. Key topics may include PI risk assessment and identification of high-risk factors, pre-transport skin assessment, protection of pressure-prone areas, positioning and repositioning strategies, prevention of medical device-related pressure injuries, as well as monitoring, documentation, and handover during transport. Such training may help healthcare providers implement appropriate PI preventive measures during transport and, in turn, improve the quality of patient care.

### 4.1 Limitations

This study has several limitations. First, it was conducted in only one province in mainland China, which may limit the representativeness and generalizability of the findings. Future studies should be undertaken across a wider range of regions to ensure greater sample diversity and broader applicability. Second, the cross-sectional design of this study precludes causal inferences regarding the relationships among PI-prevention knowledge, attitudes, and practices among prehospital emergency medical personnel involved in patient transport. Future research using longitudinal designs could better elucidate the long-term effects of factors such as training, work environment, and management strategies on PI-prevention practices. In addition, because the present study relied solely on self-reported questionnaire data, the findings may be subject to response bias. Future studies should consider incorporating direct observation and objective assessments in routine practice to further validate and refine these results.

## 5 Conclusion

This study demonstrated that prehospital emergency transport medical personnel generally exhibited positive attitudes toward PI prevention, while their knowledge base and implementation of preventive practices still have room for improvement. Participation in PI-related training was identified as a key determinant of knowledge, attitudes, and practices, with individual characteristics such as gender, profession, education, and professional title also exerting significant influence on PI prevention capability. In addition, inadequate equipment availability, limited transport space, and insufficient systemic support within the prehospital setting were found to hinder the effective implementation of PI preventive measures. These findings underscore the need to integrate PI prevention into routine training and quality management systems tailored to prehospital transport, alongside improvements in equipment provision and standardized workflows, to enhance patient safety and quality of care during transport.

## Data Availability

The datasets presented in this article are not readily available because research data will be shared with reasonable requests. Requests to access the datasets should be directed to xueyan202203@163.com.
