# Supplementary material for: Knowledge, attitudes, and practices regarding pressure injury prevention among prehospital emergency medical personnel: a cross-sectional survey
Source: Front Public Health. 2026 Apr 17;14:1781417. doi: 10.3389/fpubh.2026.1781417 (PMC13133021; doi:10.3389/fpubh.2026.1781417)
Supplement: Supplementary file 1 [file Supplementary_file_1.docx]

**Table S1 Item-level scores of the prehospital emergency medical personnel pressure injury (PI) knowledge-attitude-practice (KAP) questionnaire (*n* = 251)**

| **Dimension** | **Item** | **Question** | **Very familiar**  **n (%)** | **Fairly familiar**  **n (%)** | **Somewhat familiar**  **n (%)** | **Have heard of it**  **n (%)** | **Do not know**  **n (%)** | **Mean ± SD** |
| --- | --- | --- | --- | --- | --- | --- | --- | --- |
| **Knowledge** | 1 | Your level of familiarity with the definition of pressure injury (PI). | 57(22.7) | 115(45.8) | 74 (29.5) | 5(2.0) | 0(0.0) | 3.9 ± 0.7 |
|  | 2 | Your level of familiarity with risk factors for PI. | 67(26.7) | 115(45.8) | 61(24.3) | 5(2.0) | 3(1.2) | 3.9 ± 0.8 |
|  | 3 | Your level of familiarity with causes of PI. | 72(28.7) | 114(45.4) | 62(24.7) | 3(1.2) | 0(0.0) | 4.0 ± 0.8 |
|  | 4 | Your level of familiarity with common sites where PI occurs. | 80(31.9) | 119(47.4) | 49(19.5) | 3(1.2) | 0(0.0) | 4.1 ± 0.7 |
|  | 5 | Your level of familiarity with PI staging and the criteria used to determine stage. | 46(18.3) | 112(44.6) | 71(28.3) | 16(6.4) | 6(2.4) | 3.7 ± 0.9 |
|  | 6 | Your level of familiarity with tools used to assess PI risk. | 37(14.7) | 97(38.6) | 81(32.3) | 17(6.8) | 19(7.6) | 3.5 ± 1.1 |
|  | 7 | Your level of familiarity with preventive measures for PI. | 60(23.9) | 107(42.6) | 71(28.3) | 13(5.2) | 0(0.0) | 3.9 ± 0.8 |
|  | 8 | Your level of familiarity with key points in PI care and management. | 44(17.5) | 107(42.6) | 79(31.5) | 17(6.8) | 4(1.6) | 3.7 ± 0.9 |
|  | 9 | Your level of familiarity with medical device–related pressure injury (MDRPI). | 28(11.2) | 91(36.3) | 98(39.0) | 21(8.4) | 13(5.2) | 3.4 ± 1.0 |
| **Dimension** | **Item** | **Question** | **Strongly agree**  **n (%)** | **Agree**  **n (%)** | **Neutral**  **n (%)** | **Disagree**  **n (%)** | **Strongly disagree**  **n (%)** | **Mean ± SD** |
| **Attitude** | 1 | Most PIs that occur during transport are preventable. | 110(43.8) | 91(36.3) | 45(17.9) | 5(2.0) | 0(0.0) | 4.2 ± 0.8 |
|  | 2 | PI prevention should receive attention during long-distance transport. | 122(48.6) | 105(41.8) | 22(8.8) | 1(0.4) | 1(0.4) | 4.4 ± 0.7 |
|  | 3 | During transport, attention should be paid to populations at high risk of PI. | 132(52.6) | 101(40.2) | 18(7.2) | 0(0.0) | 0(0.0) | 4.5 ± 0.6 |
|  | 4 | I am willing to take relevant measures to prevent PI caused during transport. | 108(43.0) | 111(44.2) | 30(12.0) | 2(0.8) | 0(0.0) | 4.3 ± 0.7 |
|  | 5 | During long-distance transport, regularly checking the patient's skin condition helps prevent PI. | 130(51.8) | 98(39.0) | 21(8.4) | 1(0.4) | 1(0.4) | 4.4 ± 0.7 |
|  | 6 | PI is one of the indicators used to evaluate the quality of care during transport. | 84(33.5) | 109(43.4) | 40(15.9) | 14(5.6) | 4(1.6) | 4.0 ± 0.9 |
|  | 7 | It is very important to develop standardized PI-prevention procedures for long-distance transport. | 93(37.1) | 114(45.4) | 39(15.5) | 3(1.2) | 2(0.8) | 4.2 ± 0.8 |
|  | 8 | The attitude of transport medical personnel toward PI affects the occurrence of PI. | 91(36.3) | 106(42.2) | 35(13.9) | 14(5.6) | 5(2.0) | 4.1 ± 1.0 |
|  | 9 | PI-related knowledge training is very important. | 108(43.0) | 112(44.6) | 29(11.6) | 0(0.0) | 2(0.8) | 4.3 ± 0.7 |
|  | 10 | Participating in training helps medical personnel better prevent PI in patients. | 108(43.0) | 111(44.2) | 27(10.8) | 3(1.2) | 2(0.8) | 4.3 ± 0.8 |
|  | 11 | I proactively seek information/  knowledge related to PI. | 72(28.7) | 116(46.2) | 60(23.9) | 1(0.4) | 2(0.8) | 4.0 ± 0.8 |
| **Dimension** | **Item** | **Question** | **Always**  **n (%)** | **Often**  **n (%)** | **Sometimes**  **n (%)** | **Rarely**  **n (%)** | **Never**  **n (%)** | **Mean ± SD** |
| **Practice** | 1 | Before long-distance transport, I ask the patient or family how long the patient has maintained the same position, and based on this, choose an appropriate position at departure to help prevent PI. | 50(19.9) | 96(38.2) | 74(29.5) | 26(10.4) | 5(2.0) | 3.6 ± 1.0 |
|  | 2 | During long-distance transport, I assess PI risk to identify high-risk patients. | 49(19.5) | 96(38.2) | 62(24.7) | 33(13.1) | 11(4.4) | 3.6 ± 1.1 |
|  | 3 | During long-distance transport, I regularly check the skin at pressure points (including skin under medical devices). | 48(19.1) | 93(37.1) | 76(30.3) | 26(10.4) | 8(3.2) | 3.6 ± 1.0 |
|  | 4 | For patients at risk of PI or with existing PI, I reposition them at regular intervals. | 53(21.1) | 103(41.0) | 69(27.5) | 21(8.4) | 5(2.0) | 3.7 ± 1.0 |
|  | 5 | For patients at risk of PI or with existing PI, I use pressure-relieving measures (e.g., pressure-relieving pads/overlays or cushions). | 55(21.9) | 99(39.4) | 71(28.3) | 13(5.2) | 13(5.2) | 3.7 ± 1.0 |
|  | 6 | When temporarily securing tubing/lines, I use a bridging (“Ω”) technique to keep the tubing off the skin, thereby minimizing localized pressure and friction. | 63(25.1) | 105(41.8) | 50(19.9) | 24(9.6) | 9(3.6) | 3.8 ± 1.0 |
|  | 7 | During long-distance transport, for patients with medical devices, I regularly adjust or change the device contact sites (e.g., blood pressure cuff, pulse oximeter probe). | 55(21.9) | 108(43.0) | 55(21.9) | 24(9.6) | 9(3.6) | 3.7 ± 1.0 |
|  | 8 | During long-distance transport, for patients with medical devices, I place soft padding/support (e.g., cotton pads, foam padding) under the device to reduce pressure. | 56(22.3) | 102(40.6) | 62(24.7) | 22(8.8) | 9(3.6) | 3.7 ± 1.0 |
|  | 9 | During long-distance transport, for patients with medical devices, I keep the skin under the device clean and dry. | 61(24.3) | 110(43.8) | 59(23.5) | 16(6.4) | 5(2.0) | 3.8 ± 0.9 |
|  | 10 | During long-distance transport, I provide education to patients at risk of PI about PI prevention. | 51(20.3) | 81(32.3) | 71(28.3) | 32(12.7) | 16(6.4) | 3.5 ± 1.1 |
|  | 11 | I clearly communicate the patient's PI status and related information during handover to staff at the receiving healthcare facility. | 85(33.9) | 100(39.8) | 48(19.1) | 15(6.0) | 3(1.2) | 4.0 ± 0.9 |

**Table S2 Classification of participants according to the domain-level 80% cut-off values for PI prevention knowledge, attitude, and practice (*n*=251)**

| **Domain** | **Number of items** | **Maximum possible score** | **Cut-off value (80%)** | **Participants ≥ cut-off,**  **n (%)** | **Participants < cut-off,**  **n (%)** |
| --- | --- | --- | --- | --- | --- |
| **Knowledge** | 9 | 45 | 36 | 121(48.2) | 130(51.8) |
| **Attitude** | 11 | 55 | 44 | 172(68.5) | 79(31.5) |
| **Practice** | 11 | 55 | 44 | 109(43.4) | 142(56.6) |

Note: Cut-off values were defined as 80% of the maximum possible score for each domain. Participants with scores equal to or above the cut-off were classified as having "good knowledge", "positive attitude", or "active practice", while those below the cut-off were considered as having levels below expectation.
