# Supplementary material for: Knowledge, attitudes, and practices regarding pressure injury prevention among prehospital emergency medical personnel: a cross-sectional survey
Source: Front Public Health. 2026 Apr 17;14:1781417. doi: 10.3389/fpubh.2026.1781417 (PMC13133021; doi:10.3389/fpubh.2026.1781417)
Supplement: Supplementary file 2 [file Supplementary_file_2.docx]

**表S1院前转运急救医务人员压力性损伤知信行问卷各条目得分情况 (*n* = 251)**

| **维度** | **序号** | **问题** | **非常了解**  **n (%)** | **比较了解**  **n (%)** | **一般了解**  **n (%)** | **听说过**  **n (%)** | **不知道**  **n (%)** | **‾*χ* ± *s*** |
| --- | --- | --- | --- | --- | --- | --- | --- | --- |
| **知识** | 1 | 您对压力性损伤定义的了解程度 | 57(22.7) | 115(45.8) | 74 (29.5) | 5(2.0) | 0(0.0) | 3.9 ± 0.7 |
|  | 2 | 您对压力性损伤发生危险因素的了解程度 | 67(26.7) | 115(45.8) | 61(24.3) | 5(2.0) | 3(1.2) | 3.9 ± 0.8 |
|  | 3 | 您对压力性损伤发生原因的了解程度 | 72(28.7) | 114(45.4) | 62(24.7) | 3(1.2) | 0(0.0) | 4.0 ± 0.8 |
|  | 4 | 您对压力性损伤好发部位的了解程度 | 80(31.9) | 119(47.4) | 49(19.5) | 3(1.2) | 0(0.0) | 4.1 ± 0.7 |
|  | 5 | 您对压力性损伤的分期及其判断标准的了解程度 | 46(18.3) | 112(44.6) | 71(28.3) | 16(6.4) | 6(2.4) | 3.7 ± 0.9 |
|  | 6 | 您对压力性损伤风险评估工具的了解程度 | 37(14.7) | 97(38.6) | 81(32.3) | 17(6.8) | 19(7.6) | 3.5 ± 1.1 |
|  | 7 | 您对压力性损伤预防措施的了解程度 | 60(23.9) | 107(42.6) | 71(28.3) | 13(5.2) | 0(0.0) | 3.9 ± 0.8 |
|  | 8 | 你对压力性损伤处理要点的了解程度 | 44(17.5) | 107(42.6) | 79(31.5) | 17(6.8) | 4(1.6) | 3.7 ± 0.9 |
|  | 9 | 您对器械相关性压力性损伤的了解程度 | 28(11.2) | 91(36.3) | 98(39.0) | 21(8.4) | 13(5.2) | 3.4 ± 1.0 |
| **维度** | **序号** | **问题** | **非常同意**  **n (%)** | **同意**  **n (%)** | **持中**  **n (%)** | **反对**  **n (%)** | **非常反对n (%)** | ***‾χ* ± *s*** |
| **信念** | 1 | 转运途中绝大多数压力性损伤可避免 | 110(43.8) | 91(36.3) | 45(17.9) | 5(2.0) | 0(0.0) | 4.2 ± 0.8 |
|  | 2 | 长途转运时需要关注压力性损伤的预防 | 122(48.6) | 105(41.8) | 22(8.8) | 1(0.4) | 1(0.4) | 4.4 ± 0.7 |
|  | 3 | 转运时需要关注压力性损伤的好发人群 | 132(52.6) | 101(40.2) | 18(7.2) | 0(0.0) | 0(0.0) | 4.5 ± 0.6 |
|  | 4 | 愿意采取相关措施来预防转运途中造成的压力性损伤 | 108(43.0) | 111(44.2) | 30(12.0) | 2(0.8) | 0(0.0) | 4.3 ± 0.7 |
|  | 5 | 长途转运途中定时查看皮肤情况有利于预防压力性损伤的发生 | 130(51.8) | 98(39.0) | 21(8.4) | 1(0.4) | 1(0.4) | 4.4 ± 0.7 |
|  | 6 | 压力性损伤是衡量转运途中救护质量的指标之一 | 84(33.5) | 109(43.4) | 40(15.9) | 14(5.6) | 4(1.6) | 4.0 ± 0.9 |
|  | 7 | 制定标准化的长途转运压力性损伤预防流程非常重要 | 93(37.1) | 114(45.4) | 39(15.5) | 3(1.2) | 2(0.8) | 4.2 ± 0.8 |
|  | 8 | 转运医务人员对待压力性损伤的态度影响压力性损伤的发生 | 91(36.3) | 106(42.2) | 35(13.9) | 14(5.6) | 5(2.0) | 4.1 ± 1.0 |
|  | 9 | 压力性损伤知识培训非常重要 | 108(43.0) | 112(44.6) | 29(11.6) | 0(0.0) | 2(0.8) | 4.3 ± 0.7 |
|  | 10 | 参加培训有助于医务人员更好地预防患者压力性损伤的发生 | 108(43.0) | 111(44.2) | 27(10.8) | 3(1.2) | 2(0.8) | 4.3 ± 0.8 |
|  | 11 | 您会主动去了解有关压力性损伤的相关知识 | 72(28.7) | 116(46.2) | 60(23.9) | 1(0.4) | 2(0.8) | 4.0 ± 0.8 |
| **维度** | **序号** | **问题** | **持续**  **n (%)** | **经常**  **n (%)** | **有时**  **n (%)** | **很少**  **n (%)** | **没有**  **n (%)** | **‾*χ* ± *s*** |
| **行为** | 1 | 您会在长途转运前询问患者或其家属，该体位已持续多长时间，并据此在转运出发时选择合适体位预防压力性损伤 | 50(19.9) | 96(38.2) | 74(29.5) | 26(10.4) | 5(2.0) | 3.6 ± 1.0 |
|  | 2 | 长途转运时您会对患者进行压力性损伤风险评估筛选高危患者 | 49(19.5) | 96(38.2) | 62(24.7) | 33(13.1) | 11(4.4) | 3.6 ± 1.1 |
|  | 3 | 长途转运途中您会定期查看患者受压处皮肤情况（包括器械下面的皮肤） | 48(19.1) | 93(37.1) | 76(30.3) | 26(10.4) | 8(3.2) | 3.6 ± 1.0 |
|  | 4 | 您会对有压力性损伤风险或已患有压力性损伤的患者定期进行体位变换 | 53(21.1) | 103(41.0) | 69(27.5) | 21(8.4) | 5(2.0) | 3.7 ± 1.0 |
|  | 5 | 您会对有压力性损伤风险或已患有压力性损伤的患者使用减压设施（如减压敷料或软枕等） | 55(21.9) | 99(39.4) | 71(28.3) | 13(5.2) | 13(5.2) | 3.7 ± 1.0 |
|  | 6 | 在对患者管路进行临时固定时，您会采取“高举平抬法/Ω”以减少对局部皮肤的压力 | 63(25.1) | 105(41.8) | 50(19.9) | 24(9.6) | 9(3.6) | 3.8 ± 1.0 |
|  | 7 | 长途转运时，对使用器械的患者，您会定时更换器械使用的部位（如血压计袖带、脉氧夹等） | 55(21.9) | 108(43.0) | 55(21.9) | 24(9.6) | 9(3.6) | 3.7 ± 1.0 |
|  | 8 | 长途转运时，使用器械的患者，您会在器械下放置如棉垫、泡沫敷料等支撑物，以减轻压力 | 56(22.3) | 102(40.6) | 62(24.7) | 22(8.8) | 9(3.6) | 3.7 ± 1.0 |
|  | 9 | 长途转运时，使用器械的患者，您会保持患者器械下面的皮肤清洁干燥 | 61(24.3) | 110(43.8) | 59(23.5) | 16(6.4) | 5(2.0) | 3.8 ± 0.9 |
|  | 10 | 在长途转运过程中，您会向有压力性损伤风险的患者宣教预防压力性损伤的相关知识 | 51(20.3) | 81(32.3) | 71(28.3) | 32(12.7) | 16(6.4) | 3.5 ± 1.1 |
|  | 11 | 您会将患者的压力性损伤情况和接诊医疗机构的医护人员交接清楚 | 85(33.9) | 100(39.8) | 48(19.1) | 15(6.0) | 3(1.2) | 4.0 ± 0.9 |

**表 S2 按压力性损伤知信行各维度80%阈值划分的参与者分类 (*n*=251)**

| **维度** | **条目数** | **最高得分，分** | **阈值得分(80%)，分** | **≥ 阈值得分人数n(%)** | **< 阈值得分人数n(%)** |
| --- | --- | --- | --- | --- | --- |
| **知识** | 9 | 45 | 36 | 121(48.2) | 130(51.8) |
| **信念** | 11 | 55 | 44 | 172(68.5) | 79(31.5) |
| **行为** | 11 | 55 | 44 | 109(43.4) | 142(56.6) |

注：各维度阈值设定为该维度最高可能得分的80%。得分≥阈值者归类为“知识良好”“态度积极”或“实践积极”，低于阈值者视为未达预期水平。
